# Burnout, stress and resilience of an Australian regional hospital during COVID-19: a longitudinal study

**DOI:** 10.1186/s12913-022-08409-0

**Published:** 2022-09-02

**Authors:** Samantha J. Armstrong, Joanne E. Porter, Jo-Ann Larkins, Christopher Mesagno

**Affiliations:** 1 Victoria University, 70/104 Ballarat Rd, Footscray, VIC 3011 Australia; 2 Federation University, University Dr, Mount Helen, VIC 3350 Australia

**Keywords:** Psychological resilience, Stress resilience, Nursing, Hospital, COVID-19

## Abstract

Coronavirus disease 2019 (COVID-19) has placed huge strain on hospital staff around the world. The aim of the current longitudinal study was to investigate the resilience, stress and burnout of hospital staff located at a large, regional hospital in Victoria, Australia during the COVID-19 pandemic over time via cross-sectional surveys. The surveys were disseminated six times from August 2020 to March 2021, with the first three data collection points distributed during a state-wide lockdown. A total of 558 responses from various professional roles within the hospital over the survey period were included in the sample. Analysis of variance indicated significant main effects for the psychological variables across time, age, and workload. Hospital staff reported an increase in burnout levels throughout the eight-months. Significant negative relationships were observed between resilience and burnout, and between resilience and stress. A backward regression highlighted the contribution of resilience, stress, age, and nursing roles on burnout. Hierarchical regression analysis indicated that resilience contributed to the stress-burnout relationship. This study strengthens the evidence between resilience and burnout among healthcare workers and hospital staff and highlights the need for psychological wellbeing programs to be implemented for hospital staff impacted by a prolonged worldwide pandemic.

## Background

The year 2020 saw the declaration of the worldwide pandemic Coronavirus disease 2019 (COVID-19). By December 2021, there were 276 million recorded COVID-19 cases; almost 5.3 million deaths recorded across 222 countries and territories since the pandemic began [1] with Australia reporting over 260,000 cases and over 2000 deaths [2]. Worldwide comparisons show Australia’s COVID-19 morbidity and mortality rates are relatively low in the first year, however the pandemic placed significant strain on healthcare systems nationwide. Government mandated lockdowns (i.e., restrictions on personal active transport and socialising) to prevent the spread of COVID-19 were implemented in some Australian states. The first ‘wave’ of the COVID-19 pandemic in 2020 occurred in March/April and was accompanied by the first lockdown period in Victoria from the 31st of March to the 31st of May. The second ‘wave’ appeared in June to September and lockdown was from the 6th of August to the 9th^th^ of November and was considered to be the height of the pandemic for the year 2020 and by the end of the year, there were approximately 28,500 cases of COVID-19 [3]. The following year (2021) fluctuated with COVID-19 waves of infection, though these waves occurred outside the scope of this project. Researchers have shown that lockdowns result in poorer mental health for individuals worldwide [4–12] and also healthcare workers [13]. Australian populations have also suffered psychologically from the enforced lockdowns [14–21], including those within the health care system such as nurses, physicians, and allied health staff [22].

Burnout and stress are familiar terminology and often used synonymously, especially during COVID-19. Stress is defined as any non-specific demand that can affect a person’s physiological and psychological bodily processes, resulting in our ability or inability to cope and can lead to psychophysiological vulnerability or thriving [23]. Burnout is the accumulation of stress over time and is characterised by feelings of mental and physical exhaustion, negative attitude, and feeling like workplace goals are unachievable [24–26]. Before the COVID-19 pandemic, a review of Australian hospital (nursing) staff highlighted moderate to high levels of stress and burnout [27], particularly staff working in emergency departments [28, 29]. Burnout is more prominent in younger populations within hospital settings [30]. Staff in metropolitan hospitals were also more likely to suffer from symptoms of stress and burnout compared to regional hospitals [31, 32].

During the COVID-19 pandemic, hospital staff, including physicians, nurses, administration, and human resources were under pressure to prepare and manage the personal and occupational consequences of COVID-19. Hospital staff, particularly frontline staff (i.e., working in the COVID-19 hospital wards) and emergency department personnel [33, 34], were at a higher risk of contracting COVID-19 compared to the general population [35–37]. In Australia, healthcare workers were subjected to three times the risk of infection compared to the general population during the first 6 months of the pandemic [38]. Victoria had the highest infection rates when compared to other states during the second wave of the virus (August 2020), which saw 3500 healthcare worker infections [39, 40]. In response to COVID-19, some hospitals within Australia became designated COVID-19 hospitals, with any person suspected of, or confirmed to have, COVID-19 transported to a COVID-19 hospital. As COVID-19 symptoms are similar to many other illnesses (e.g., influenza), the caseload for hospital staff significantly increased for potential COVID-19 infected persons. This contributed to the strain on the healthcare system, and in addition significantly impacted the health and wellbeing of hospital staff.

Multiple factors contributed to poor psychological wellbeing of hospital staff. For both clinical and non-clinical staff, COVID-19 forced changes to procedural and working conditions such as the introduction of retraining programs, which increased staff workload [41]. Hospital staff contended with the fear of virus transmission to family members [42–44] and a limited availability of personal protective equipment [45]. The closure of education centres, such as schools and pre-school learning centres, meant healthcare workers with children could no longer work their regular employment hours [46]. Similar to other countries, the COVID-19 changes adversely affected the mental health of hospital staff resulting in increased levels of stress, anxiety, depression and burnout [42, 47–51], particularly frontline hospital staff [48, 52] and nurses [48, 53, 54]. Medical/clinical healthcare personnel demonstrated poorer mental health outcomes in comparison to non-medical healthcare personnel during COVID-19 [55, 56].

Poor mental health as a consequence of the pandemic prompted further government initiatives to promote positive psychological and physiological health and wellbeing within the workplace such as the Healthcare Worker Infection Prevention and Wellbeing Program implemented in November 2020. One of the aims of the health and wellbeing programs was to build personal resilience among the workforces. Whilst resilience has become a ‘buzzword’ in recent years, its importance has never been more pertinent in a time of a pandemic. Whilst the operational definition of stress resilience is contentious, researchers propose that stress resilience emphasises both the psychological and physiological stress processes that encourages positive and/or negative adaptations in the face of adversity, which can lead to optimised psychophysiological functioning or psychophysiological vulnerability [57–60]. An individual’s level of stress resilience is founded upon their adaptability to the current situation and based on what they have learned from previous experience [61]. An individual’s resilience, stress, and burnout levels are practically and theoretically dependent. Researchers found that hospital personnel with high levels of resilience are more able to manage and overcome workplace stress [62–65]. Additionally, individuals that indicate lower levels of stress and moderate to high levels of resilience are less likely to suffer from burnout [66, 67]. In addition, individuals that suffer from burnout are more likely to consider job resignation [68] and hospital staff that present with greater resilience show better workplace longevity [69, 70]. Researchers have suggested that older individuals are more resilient to occupational stress [71] and COVID-19-related stressors [22, 51]. One possible reason for these results might be that greater workplace experience is linked to greater resilience [72]. Thus, as age increases, exposure to workplace stressors increase, which may help develop psychological resilience. Peripherally, age appears to be an optimising factor for resilience. Furthermore, workload can influence stress and burnout; hospital staff that work long hours exhibit higher stress and their feelings of resilience are limited in comparison to staff working less hours [73]. Workload is positively correlated with burnout [74, 75].

Whilst it is apparent that literature on stress, burnout and resilience amongst hospital- based health care workers (mainly physicians and nurses) is well researched, there appears to be limited investigation conducted on other workplace roles within these hospitals. Quantitative research that aims to contribute to the research lacuna and complement the existing data is warranted. Longitudinal research on COVID-19 is limited [76], with few time-series studies observing the effect of COVID-19 on the psychological wellbeing of healthcare workers [77–79], and minimal studies focused on Australian health workers. Therefore, collecting time-series data from hospital staff during a worldwide pandemic working from a regional, designated COVID-19 hospital over time can inform on the mental health of hospital staff for future pandemics. This paper will present findings of an eight-month stress resilience study within a large, regional hospital.

## Methods

### Participants

Participants were recruited from a large, regional hospital in Victoria, Australia and included staff across multiple divisions, including people and culture, clinical services, high acuity services, medical services, mental health services, education and training and information and regional services. A total of 648 responses were submitted across the six surveys and after data cleaning yielded a cumulative total of 558 hospital staff submissions that gave usable responses in the surveys. Declining response rates occurred over the six data collection points, with the surveys yielding 137 (August), 141 (September), 95 (October), 68 (November), 54 (December) and 63 (February/March) completed responses. Given an estimated hospital workforce available at time of sampling of 2000 employees, a power analysis suggested sample sizes of between 66 (at 90% confidence with a 10% margin of error) to 323 (at 95% confidence with a 5% margin of error). The number of responses for each sampling event are compatible with this range of estimates.

Overall, the sample across all surveys was female dominant (453), with 98 males, and seven participants that preferred to not say. Staff over the age of 40 made up 59.3% of the sample. For analysis, the participants that indicated their professional position within their workplace were split into three groups: nursing (emergency, midwifery), medical (physicians, anaesthetists), and other (all non-medical and non-nursing staff). Based on aggregated participant categories, data showed there were mostly nurses completing the surveys (243), although the other groups were relatively evenly spread (medical = 132, other = 152). The sample were mainly full-time hospital staff (407, 72.9%) with the remainder of participants working part-time or casually employed (27.1%). The clinical services and mental health departments were the most engaged throughout data collection (299 submissions). Professional longevity within the workforce showed staff that had six or more years’ experience in the field (46.9%) had the greatest engagement across the surveys, compared to staff who had two to 6 years’ experience (25.3%), and less than 2 years’ experience (27.5%) within their profession.

### Measures

Basic demographic information included information of participants such as gender, age, professional role within the workplace, workload, and workplace longevity at the current hospital.

#### Resilience

The Brief Resilience Scale (BRS; [80]) is a 6-item questionnaire designed to assess an individual’s ability to recover from stressful circumstances [81]. Questions include *I tend to bounce back quickly after hard times,* and *I usually come through difficult times with little trouble*. Answers are provided on a five-point Likert scale ranging from 1 (*strongly disagree*) to 5 *(strongly agree*). Since the total is divided by the total number of items, the combined scores range from 1 to 5, with scores from 1.00–2.99 indicating low resilience, 3.00–4.30 moderate resilience, and 4.31–5.00 high resilience [82]. The scale displays acceptable internal consistency (a = .80–.91; Smith et al., 2008) and has been used internationally with psychometric support [81]. Test-retest reliability is adequate with an intraclass correlation of .69 over 4 weeks with 48 participants and .62 for 12 weeks with 61 participants [80]. Reliability analyses for the current sample were acceptable with a Cronbach’s a score of .86.

#### Stress

Stress was assessed with the Perceived Stress Scale (PSS; [83]), which is a 10-item questionnaire assessing an individual’s level of stress within their current situation and feelings of control, including daily stressors to major events over the past month. An example question is, *In the last week, how often have you been upset because of something that happened unexpectedly?* Answers are provided on a five-point Likert scale ranging from 0 (*never*) to 4 *(very often*). Items four, five, seven and eight are reverse scored, and the 10 items are summed for a total score. Scores range from 0 to 40 with higher scores indicating higher stress. Scores from 0 to 13 indicating low stress, 14–26 moderate stress, and 27–40 high stress levels. The PSS has good psychometric properties showing strong test-retest reliability (*r* = .90 for a two-week interval [84];), good internal consistency [85], and adequate convergent and discriminant validity with other stress inventories [86]. Reliability analyses for the current sample were acceptable with a Cronbach’s alpha score of .87.

#### Burnout

The 14-item Shirom-Melamed Burnout Measure (SMBM; [87]), a shortened version of the Shirom-Melamed Burnout Questionnaire [88], was used to assess symptoms of occupational burnout. Burnout is measured on three subscales: physical fatigue, emotional exhaustion, and cognitive weariness. Questions include *I am physically drained*, and *my thinking process is slow*. Minor changes were made to four questions on the SMBM. SMBM 4 wording was changed from ‘dead’ to ‘flat’ since consideration was given for emergency personnel managing hospital mortality. SMBM wording for questions 12, 13 and 14 was changed from “customers” to “patients” since using patients is better aligned with their workplace interactions. Items were measured on a Likert scale from 1 (*almost never*) to 7 (*almost always*). The SMBM scores were represented as the average of the 14 total items with higher scores reflecting high symptoms of burnout. The SMBM shows adequate internal consistency with majority of studies scoring a = > 0.70 [87, 89–91]. Regarding construct validity, the SMBM is well correlated with other reliable burnout measures, such as the Maslach Burnout Inventory and the Shirom-Melamed Burnout Questionnaire [88, 90]. Reliability analyses for the current sample were acceptable with a Cronbach’s alpha score of .96.

### Procedure

Emails to participate in the study were facilitated by the Education and Research facility at the regional hospital. The email contained an electronic link to the online survey. The survey comprised of a plain language information statement and by agreeing to complete and submit the survey, the participant agreed to full consent. Once the participant’s survey was submitted, the data was unable to be withdrawn since all data collected was anonymous. The survey took 10 minutes to complete.

**Fig. 1:**
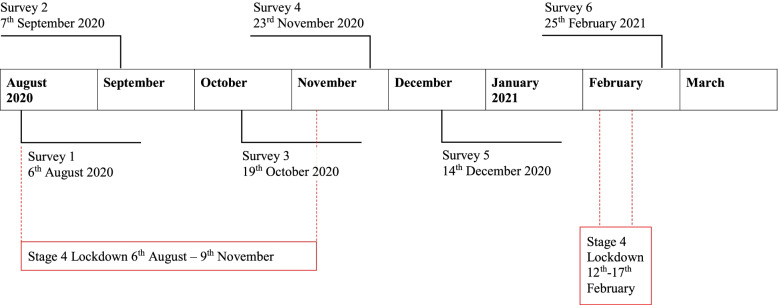
Timeline of study. Red dotted line denotes time in lockdown

The surveys were disseminated by the director of research at the regional hospital to all staff members each month from August 2020 to March 2021 (with the exception of January). Each survey was accompanied by one reminder email before the closure date. There were six data extraction points over an eight-month period. The participants that chose to participate in each of the monthly surveys were submitted anonymously, and therefore participants could not be ‘tracked’ throughout the six data collection time-points. The months of February and March were combined due to low response rates in those months. Each survey was open for 1 week, with the exception of the last survey, which was open for 2 weeks across February and March. The first, second and third surveys were disseminated during the second government mandated lockdown period in Victoria, Australia. Subsequent surveys were conducted outside of the lockdown period. The beginning of 2021 suggested that the contagion level of COVID-19 within Australia was declining and therefore the study concluded survey distribution after the sixth survey (see Fig. 1 for survey dissemination timeline).

### Data analysis

Descriptive analysis was conducted to understand demographical trends on the main variables. A one-way, between groups analysis of variance (ANOVA) was conducted to examine the changes in resilience, stress, and burnout over time (between groups variable). A multifactorial ANOVA was used to determine the impact of age, gender, workload, professional longevity, and work role within the hospital upon the dependent variables of resilience, stress, burnout and time. Spearman’s correlation coefficients were calculated to examine the relationships between variables. Backward multiple regression was used to assess significant factors that contributed to burnout. Finally, a hierarchical multiple regression was conducted to observe the mediating role of resilience on burnout. All statistical analyses were computed using SPSS (Version 26.0). Alpha was set at *p* < .05 significance for all analyses and where applicable partial eta squared (partial η^2^) was used to measure effect sizes.

## Results

### Data cleaning

To manage missing data, a modified listwise deletion method was implemented, deleting completely random cases with more than one test battery incomplete, rather than one or more missing value. Whilst Miettinen [92] suggested the latter method is the only approach to assure no bias has been introduced, Vach [93] postulates the draconian rules of listwise deletion limit the scope of the data and the method should be more reasoned and fluid, hence resulting in a modified data cleaning method. Cases removed (by data time point) from the total sample of 648 included: 25 (August), 31 (September), 9 (October), 15 (November), 1 (December), and 7 (February/March). Mean replacement was not used for missing values as the missing item guidelines were exceeded on those occasions.

### Analysis of variance

#### Time

**Fig. 2:**
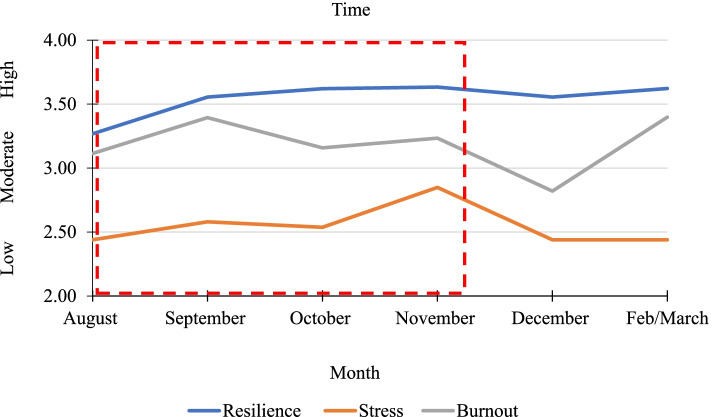
Mean scores for resilience, stress and burnout over time. Red dotted line denotes time in lockdown

Table 1 presents the means and standard deviations for resilience, stress and burnout over the six time points. For each of the six surveys, resilience and burnout scores indicate moderate levels that are comparable to general population norms [80, 90]. Stress scores for the sample indicate moderate to high levels of stress [83]. Figure 2 shows the mean scores over time with corresponding lockdown periods.

**Table 1:** Means and standard deviations for resilience, stress, and burnout across time

Time	Resilience				Stress				Burnout			
	***M***	***SD***	***Min***	***Max***	***M***	***SD***	***Min***	***Max***	***M***	***SD***	***Min***	***Max***
August	3.25	.69	1.83	5.00	24.30	6.57	10.00	40.00	3.14	1.14	1.00	6.93
September	3.52	.71	1.50	5.00	25.87	7.21	10.00	40.00	3.42	1.22	1.00	6.93
October	3.61	.69	2.00	5.00	25.02	6.77	10.00	40.00	3.10	1.25	1.21	6.86
November	3.65	.74	2.00	5.00	28.62	3.08	22.00	36.00	3.25	1.22	1.43	6.57
December	3.55	.58	2.33	5.00	23.94	6.50	11.00	39.00	2.87	1.04	1.00	5.64
February/March	3.58	.71	2.00	5.00	25.41	7.03	11.00	40.00	3.50	1.18	1.43	6.93

The ANOVA showed a main effect for time and resilience, *F* (5, 505) = 4.09, *p* < .001, with a small Cohen [94] effect size (partial η^2^ = .04). Post-hoc comparisons using Tukey HSD indicated significant differences for August, indicating significantly lower resilience compared to all other data collection times. A significant main effect was evident for time and stress, *F* (5, 502) = 4.34, *p* < .001, partial η^2^ = .04. The month of November saw the highest stress scores compared to other data collection months with Tukey HSD identifying November significantly different from all months except February/March. A significant main effect was also found for burnout and time, *F* (5, 509) = 2.50, *p* < .05, partial η^2^ = .03. Hospital staff exhibited significantly higher scores for burnout for September compared to December data collection period, but no other significant differences were found.

#### Age

Table 2 shows the means and standard deviations for age across resilience, stress and burnout parameters. The ANOVA showed a main effect for age and resilience, *F* (6, 505) = 3.12, *p* < .005, partial η^2^ = .04. Significant differences on resilience scores were found for the 26–30 age bracket in comparison to the 31–35 age bracket, the 36–50 age bracket, the 41–50 age bracket and the 61–70 age bracket, but not the 21–25 age bracket or 51–60 age bracket showing the lower age group exhibiting lower resilience scores. A main effect was found for age and stress, *F* (6, 502) = 3.12, *p* < .005, partial η^2^ = .04, whereby hospital staff in their low 30s [31–35] showed significantly higher scores on stress compared to staff aged 36 and above. A significant age main effect was found for age and burnout, *F* (6, 509) = 6.35, *p* < .001, partial η^2^ = .07, highlighting that staff aged 31–35 showed greater burnout scores compared to the 26–30 age bracket, the 36–40 age bracket, the 41–50 age bracket, the 51–60 age bracket and the 61–70 age bracket, although not the 21–25 age bracket.

**Table 2:** Means and standard deviations for age across resilience, stress, and burnout parameters

Age	Resilience		Stress		Burnout	
	*M*	*SD*	*M*	*SD*	*M*	*SD*
21–25 (*n* = 33)	3.28	.70	27.85	6.73	3.5	1.22
26–30 (*n* = 71)	3.22	.69	26.33	6.63	3.32	1.03
31–35 (*n* = 66)	3.54	.77	27.85	7.11	3.99	1.40
36–40 (*n* = 55)	3.58	.56	24.51	6.70	3.26	1.16
41–50 (*n* = 128)	3.58	.74	24.80	6.84	3.11	1.14
51–60 (*n* = 153)	3.45	.71	24.95	6.11	3.04	1.16
61–70 (*n* = 50)	3.69	.58	23.68	5.70	2.80	.94

#### Workload

The ANOVA showed a main effect for workload and resilience, *F* (5, 505) = 5.02, *p* < .001, partial η^2^ = .05, with higher resilience scores for hospital staff at a higher workload capacity. Whilst all staff indicated a moderate level of resilience across different workloads, a significant difference was evident between full-time staff (*M* = 3.65, *SD* = 0.71) and staff working .4 EFT (*M* = 3.27, *SD* = 0.64), .6EFT (*M* = 3.33, *SD* = 0.72) and .8EFT (*M* = 3.53, *SD* = 0.66), respectively. No significant results were found for stress, *F* (5, 502) = .87, *p* > .05, or burnout, *F* (5, 490) = .95, *p* > .05, across workload.

#### Workplace position

The ANOVA indicated no main effects for workplace position for resilience, *F* (2, 505) = .04, *p* > .05, stress, *F* (2, 502) = 1.27, *p* > .05, or burnout, *F* (2, 490) = .30, *p* > .05.

### Correlations

A Spearman’s bivariate correlational analysis was conducted to explore the relationships between age, workload, resilience, stress, and burnout (Table 3). There was a small, significant positive relationship between age and resilience, *rho* = .14, *n* = 556, *p* < .01. Significant negative relationships were found for age and stress, *rho* = .14, *n* = 553, *p* < .01., and age and burnout, *rho* = .19, *n* = 539, *p* < .01, although both relationships indicated weak associations according to Cohen [94]. Significant, weak positive relationships were prevalent for workload and resilience, *rho* = .20, *n* = 556, *p* < .01. Moderate, negative associations were observed between resilience and stress, *rho* = -.30, *n* = 555, *p* < .01 and resilience and burnout, *rho* = -.36, *n* = 541, *p* = .01. The strongest, positive relationship was evident between stress and burnout, *rho* = .58, *n* = 541, *p* < .01.

**Table 3:** Correlation matrix (Spearman) for gender, age, workload, position, resilience, stress and burnout

	Gender	Age	Workload	Position	Resilience	Stress	Burnout
Gender	–	-.13** (*n* = 550)	-.17** (*n* = 549)	-.13** (*n* = 520)	-.08* (*n* = 551)	.01 (*n* = 548)	.06 (*n* = 534)
Age		–	-.01 (*n* = 554)	.14** (*n* = 524)	.14** (*n* = 556)	-.14** (*n* = 553)	-.19** (*n* = 539)
Workload			–	.24** (*n* = 526)	.20** (*n* = 539)	-.04 (*n* = 553)	-.05 (*n* = 539)
Position				–	.05 (*n* = 526)	.03 (*n* = 523)	-.09* (*n* = 511)
Resilience					–	-.30** (*n* = 555)	-.36** (*n* = 541)
Stress						–	.58** (*n* = 540)
Burnout							–

* *p* < .05 (two-tailed); ** *p* < .01 (two-tailed)

### Regressions

A backward multiple regression analysis was conducted to determine which variables significantly contributed to burnout (Table 4). The variables age, gender, workload, position within the hospital (medical and nursing dummy variables), stress, and resilience were entered into the model and explained 38.3% of the variance toward burnout, *R*^2^ = 383, adjusted *R*^2^ = .374, *F* (7, 485) = 42.95, *p* < .001. Step 2 removed gender from the model, and Step 3 removed medical position from the model with both steps explaining the same variance percentage as Step 1. Step 4 removed workload explaining 38.1% of the variance towards burnout, *R*^2^ = 381, adjusted *R*^2^ = .376, *F* (4, 488) = 75.01, *p* < .001. Unstandardised (*B*) and standardised (*ß*) regression coefficients, and square semi-partial or ‘part’ correlations (*sr*^2^) for each predictor are reported in Table 4.

**Table 4:** Results of backward method standard regression analysis (Dependent Variable- Burnout)

	Step 1						Step 2					Step 3					Step 4			
		CI_95%_ For *B*					CI_95%_ For *B*					CI_95%_ For *B*					CI_95%_ For *B*			
Variable	*B*	Lower	Upper	*ß*	*sr*^*2*^	*B*	Lower	Upper	*ß*	*sr*^*2*^	*B*	Lower	Upper	*ß*	*sr*^*2*^	*B*	Lower	Upper	*ß*	*sr*^*2*^
BRS	-.28**	-.40	-.15	-.16	-.15	-.28**	-.40	-.15	-.16	-.15	-.28**	-.40	-.15	-.16	-.15	-.26**	-.38	-.14	-.15	-.15
PSS	.10**	.08	.11	.01	.51	.10**	.08	.11	.53	.51	.10**	.08	.11	.54	.91	.10**	.08	.11	.54	.51
Age	-.06*	-.10	-.01	-.08	-.08	-.06*	-.10	-.01	-.08	-.08	-.06*	-.10	-.01	-.08	-.08	.06*	-.12	-.01	-.09	-.08
Nursing	.20	-.01	.40	.08	.07	.20	-.01	.40	.08	.07	.18*	.01	.35	.08	.07	.16	-.01	.33	.07	.07
Workload	.05	-.03	.14	.06	.04	.05	-.03	.14	.05	.04	.05	.14	-.02	.05	.04					
Medical	.03	-.20	.26	.01	.01	.03	-.20	.26	.01	.01										
Gender	-.00	-.26	.22	.00	.00															
*R*	.62					.62					.62					.62				
*R*^*2*^	.38					.38					.38					.38				
?*R*^*2*^	.38**					.00					.00					.00				

*N* = 508

*CI* Confidence interval

* *p* < .05; ** *p* < .00. *BRS* Brief Resilience Scale; *PSS* Perceived Stress Scale

## Discussion

The purpose of this study was to observe the psychological wellbeing of Australian regional hospital staff across six data time points over eight months of the COVID-19 pandemic. The primary aims were to examine psychological parameters of hospital staff and to provide insight on the health-related consequences of COVID-19 over time related to resilience, stress and burnout and the contribution of resilience and stress on burnout.

### Burnout’s crescendo

Based on the unprecedented chronic nature of COVID-19, it is not surprising that hospital staff burnout rates increased during this longitudinal study. Despite the low mortality rates in Australia compared to other countries, the psychological wellbeing of hospital staff is in peril. The increasing rates of burnout symptoms may be attributed to fear of contagion [95], perception of workplace support [96], or prolonged anticipation of a disaster in a constantly changing environment [97], suggesting a constant state of psychological alertness and fear of the high mortality rates among healthcare workers globally [98]. Since these attributions are largely speculative, more research is necessary to determine the most accurate cause.

### Associations with COVID-19 lockdown

It was presumed that high stress and burnout symptoms would parallel with the COVID-19 lockdown time periods. This was partially supported. Firstly, burnout scores were similar across the three and a half months of lockdown, with September (middle of lockdown) showing the highest scores for burnout of hospital staff. There were differences in burnout scores between September and December, providing a comparison between lockdown and non-lockdown periods. These results are similar to Smallwood et al’s [22] cross-sectional study on 9518 Australian healthcare workers that coincided with the second Melbourne lockdown (September to October) who found participants with high scores in resilience still experienced high burnout. Yet the current study’s burnout scores were less severe. Smallwood et al. suggested that resilience may not assist in protecting individuals from psychological vulnerability during COVID-19, which corresponds with the current results that resilience had a small but worthy contribution towards burnout compared to stress. November burnout scores were similar to scores during lockdown period. Unexpectedly, the highest burnout scores were seen during the months of February/March, at the end of the data collection period. When this study was initially developed, the extended duration of this pandemic was not considered, and emphasis was on lockdown periods having the greatest impact on stress and burnout. In hindsight, the prolonged duration of the pandemic has meant healthcare workers are enduring chronic states of workplace burnout. Speculatively, that may be why burnout scores were high during the last survey. Smallwood et al. [22] concluded that the moderate to severe burnout rates across healthcare workers in Australia are not surprising considering the prolonged duration of the pandemic coupled with the multiple, enforced lockdown restrictions. Secondly, for stress, significant differences were seen between lockdown and non-lockdown periods, with November (a non-lockdown period) indicating the highest stress scores, while August and October (during lockdown) showing lower stress scores. Two small cross-sectional studies conducted outside of lockdown in metropolitan Melbourne hospital staff during COVID-19 (from April to June 2020) indicated low to moderate levels of stress [99] and burnout [65]. Based on the timeline of the aforementioned studies, and the current study’s data collection timeline, an accumulative effect upon stress levels for hospital staff and healthcare workers may have occurred; as the pandemic duration increases, stress increases potentially contributing to an increased rate of burnout.

### Correlations

It was hypothesised that there would be a negative correlation between resilience and stress and resilience and burnout. As expected, there were significant moderate, negative associations between resilience and stress, and resilience and burnout. The observed relationships and strength between variables are consistent with previous findings on nursing populations [67, 100–103]. Furthermore, as age increased, resilience also increased across the time points, complementing past research [22, 51, 71]. Although, no significant findings were exhibited for age on stress and burnout for the current study. This is contradictory to past research which highlights a significantly higher prevalence of burnout for younger nursing staff under 30 years of age [104]. A meta-analysis by Brewer and Shapard [105] showed a strong positive correlation between age and burnout which was not evident in our current results.

### Staff workload during a pandemic

It was presumed that hospital staff with a greater workload would indicate higher stress and burnout with corresponding lower resilience levels. Contradictorily, hospital staff with a higher workload showed significantly greater resilience than staff working part-time. This finding is inconsistent with other research [73] that found long hours and shift work negatively impacted their personal resilience, although this research was not conducted during a pandemic. Further correlational analyses indicated age and level of experience were evenly distributed across workload classifications and therefore did not contribute valuable information as to why the hypothesis was not supported. A cross-sectional study on the experiences of Australian nurses during COVID-19 indicated that there was a decrease in work hours and clinical tasks during the height of COVID-19 [106]. This may account for the current study results, whereby full-time staff may have experienced a reduced workload, indicating why greater resilience was apparent for full-time workers. Part-time staff are more likely to have young families [107] and the closure of schools led to children completing their schoolwork from home. Home schooling may have increased the workload for part-time hospital staff and may also suggest why their resilience levels were significantly lower than their full-time colleagues. In addition, individuals working part-time may have normally used their spare time to engage in leisure and social activities, which has been shown to improve psychological wellbeing [108, 109], but since these activities were limited during lockdown, this may have affected part-time staff resilience levels.

### Clinical versus non-clinical

It was expected that clinical hospital staff (nurses and physicians) would indicate greater stress and burnout compared to other hospital staff members. Contrary to the hypothesis, there were no statistically significant differences among hospital staff for resilience, stress and burnout. A recent study on healthcare workers during COVID-19 found no differences between physician or nurse’s levels of stress (or depression) and in addition, no associations were identified between poor mental health outcomes and staff involved in treatment of COVID-19 patients in comparison to staff involved in other non-COVID-19-related hospital duties [51]. This is consistent with additional research on professional roles of hospital staff (clinical or other) during COVID-19 [65]. The current results suggest that regardless of position within the hospital, and despite direct involvement with COVID-19 patients, hospital staff as a group experience similar rates of stress and burnout. All staff may interact with a COVID-19 patient, have a fear of contagion, and the limitation of social support due to implemented lockdowns may contribute to stress and burnout, regardless of their professional role within the hospital workplace.

### Limitations

Whilst the current findings present a snapshot of hospital staff during COVID-19, there are limitations that must be considered when drawing conclusions. Firstly, the study was cross-sectional therefore difficult to interpret the data changes ‘across time’ since we could not track within-subjects data throughout the six data collection points. Ideally, a repeated-measures within-subjects design across six time-points would have generated more informative data sets regarding interpretation ‘over time’. Though, this was not possible with the current sample. Secondly, the declining, modest response rates throughout the data collection time points temper conclusions regarding the representativeness of the current findings. Lower response rates may have been due to survey fatigue. Lastly, due to the unexpected nature of a healthcare disaster, we were unable to obtain baseline data to compare before COVID-19 began, but instead, data could be collected post-COVID-19 to determine the resilience, stress, and burnout levels when the COVID-19 threat subsides (when vaccination rates increase).

### Implications

The findings of this study present additional avenues for further research. Because stress resilience is a multidimensional construct, it is important to determine the core components of stress resilience and how it is then reflected and measured within the research. In addition, the current study assessed the contributory effect of resilience on burnout using time-point cross-sectional data, thus future research should consider a within-subjects longitudinal study as this will strengthen the assumptions of resilience contributing to psychological optimisation. Research during a pandemic should also obtain further personal participant information to better inform further contributory factors that may impact psychological wellbeing such as, family situation, financial distress, and any pre-existing mental health conditions. In addition, a more extensive examination of workplace roles during a pandemic (compared to regular professional roles before a pandemic) would provide further insight on the impact of a pandemic on individuals working within the hospitals. Within a pandemic situation, it would be useful to compare a designated COVID-19 hospital with a non-COVID-19, creating a potential control group for comparison.

## Conclusion

Whilst mindful of the cross-sectional design of the current study, hospital staff showed a moderate level of burnout throughout the six data collection points of this study, though data shows symptoms of burnout are steadily increasing. Due to a lack of longitudinal research, it is unknown whether the psychological health of Australian healthcare workers is worsening, yet it can be assumed that the healthcare population will follow similar global trends presenting poor mental health outcomes as time progresses. Hospital staff showed high stress during the month of November, yet thankfully other data collection time-points showed moderate levels of stress. Additionally, the current data contends younger hospital staff are at a greater risk of burnout which is concerning as younger hospital staff in the current study showed lower resilience compared to older staff working a part-time load. Hospital staff would benefit from supportive interventions for the current pandemic and during future healthcare crises and strategies attempting to improve the psychological health of hospital staff could target younger populations. Resilience training programs may assist in the prevention of workplace burnout and psychosocial interventions may assist with halting the decline of burnout of hospital workers during COVID-19. Further longitudinal data during and post-COVID-19 is required to ascertain the effect of a pandemic on the psychological health of our sorely needed healthcare professionals and hospital staff.

## Data Availability

The data that support the findings of this study are available from Latrobe Regional Hospital, but restrictions apply to the availability of these data which were used under license for the current study, and so are not publicly available. Data are however available from the authors upon reasonable request and with permission of Latrobe Regional Hospital.

## Abbreviations

BRS: Brief Resilience Scale
COVID-19: Corona-virus disease 2019
PSS: Perceived Stress Scale
SMBM: Shirom-Melamed Burnout Measure
